# 4-Dimethyl­amino-*N*′-(4-nitro­benzyl­idene)benzohydrazide methanol monosolvate

**DOI:** 10.1107/S1600536812037063

**Published:** 2012-09-01

**Authors:** Xiyue Zhang, Xiaobo Fu, Langzhu Tan, Chaixia Wang, Weixing Fan

**Affiliations:** a China Animal Heath and Epidemiology Center, Qingdao 266032, People’s Republic of China; b Worker Hospital of Qingdao Salt Industry, Qingdao 266012, People’s Republic of China; c University of Pittsburgh School of Medicine, Pittsburgh, PA 15261, USA

## Abstract

In the title compound, C_16_H_16_N_4_O_3_·CH_3_OH, the aromatic rings form a dihedral angle of 0.4 (2)°. The nitro group is twisted from the attached benzene ring by 7.5 (2)°. In the crystal, N—H⋯O and O—H⋯O hydrogen bonds link alternating hydrazone and methanol mol­ecules into chains in [100]. The crystal packing exhibits π–π inter­actions between aromatic rings from neighbouring chains [centroid–centroid distances = 3.734 (3) and 3.903 (3) Å].

## Related literature

For the biological activity of hydrazone compounds, see: Zhang *et al.* (2012 ▶); Cacic *et al.* (2006 ▶); Rauf *et al.* (2008 ▶); Bedia *et al.* (2006 ▶). For similar hydrazone compounds, see: Horkaew *et al.* (2012 ▶); Kargar *et al.* (2012 ▶); Hu & Liu (2012 ▶). For reference bond lengths, see: Allen *et al.* (1987 ▶).
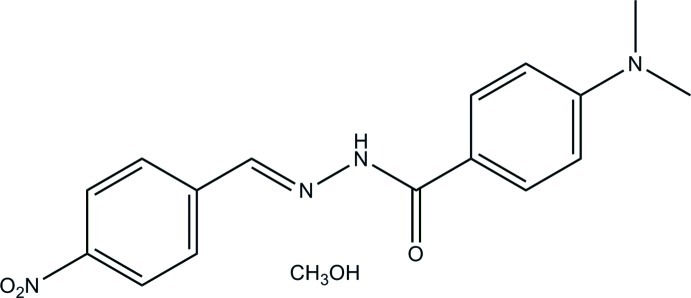


## Experimental

### 

#### Crystal data

C_16_H_16_N_4_O_3_·CH_4_O
*M* *_r_* = 344.37
Triclinic, 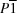

*a* = 6.6621 (12) Å
*b* = 10.6685 (17) Å
*c* = 13.3437 (13) Å
α = 72.279 (2)°
β = 83.444 (2)°
γ = 73.984 (2)°
*V* = 867.9 (2) Å^3^
*Z* = 2
Mo *K*α radiation
μ = 0.10 mm^−1^
*T* = 298 K
0.30 × 0.27 × 0.23 mm

#### Data collection

Bruker SMART CCD area-detector diffractometer
Absorption correction: multi-scan (*SADABS*; Sheldrick, 1996) ▶ *T*_min_ = 0.972, *T*_max_ = 0.978
6198 measured reflections
3140 independent reflections
2082 reflections with *I* > 2σ(*I*)
*R*_int_ = 0.035

#### Refinement

*R*[*F* ^2^ > 2σ(*F* ^2^)] = 0.058
*wR*(*F* ^2^) = 0.208
*S* = 1.08
3140 reflections
233 parameters
1 restraint
H atoms treated by a mixture of independent and constrained refinement
Δρ_max_ = 0.32 e Å^−3^
Δρ_min_ = −0.40 e Å^−3^

### 

Data collection: *SMART* (Bruker, 1998 ▶); cell refinement: *SAINT* (Bruker, 1998 ▶); data reduction: *SAINT*; program(s) used to solve structure: *SHELXS97* (Sheldrick, 2008 ▶); program(s) used to refine structure: *SHELXL97* (Sheldrick, 2008 ▶); molecular graphics: *SHELXTL* (Sheldrick, 2008 ▶); software used to prepare material for publication: *SHELXTL*.

## Supplementary Material

Crystal structure: contains datablock(s) global, I. DOI: 10.1107/S1600536812037063/cv5333sup1.cif

Structure factors: contains datablock(s) I. DOI: 10.1107/S1600536812037063/cv5333Isup2.hkl

Supplementary material file. DOI: 10.1107/S1600536812037063/cv5333Isup3.cml

Additional supplementary materials:  crystallographic information; 3D view; checkCIF report

## Figures and Tables

**Table 1:** Hydrogen-bond geometry (Å, °)

*D*—H⋯*A*	*D*—H	H⋯*A*	*D*⋯*A*	*D*—H⋯*A*
O4—H4⋯O3	0.82	1.92	2.733 (2)	170
N2—H2⋯O4^i^	0.89 (1)	2.01 (1)	2.889 (2)	170 (2)

Symmetry code: (i) 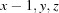.

**Fig. 1.**
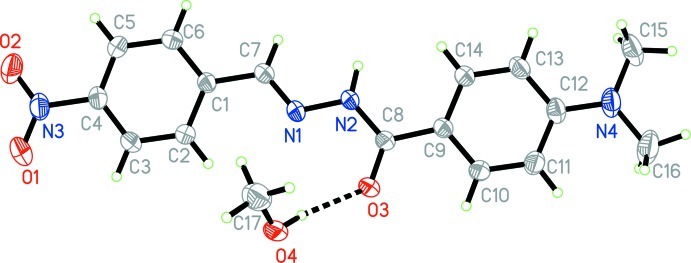
Molecular structure of the title compound showing the atomic numbering and 30% probability displacement ellipsoids. Hydrogen bond is drawn as a dashed line.

**Fig. 2.**
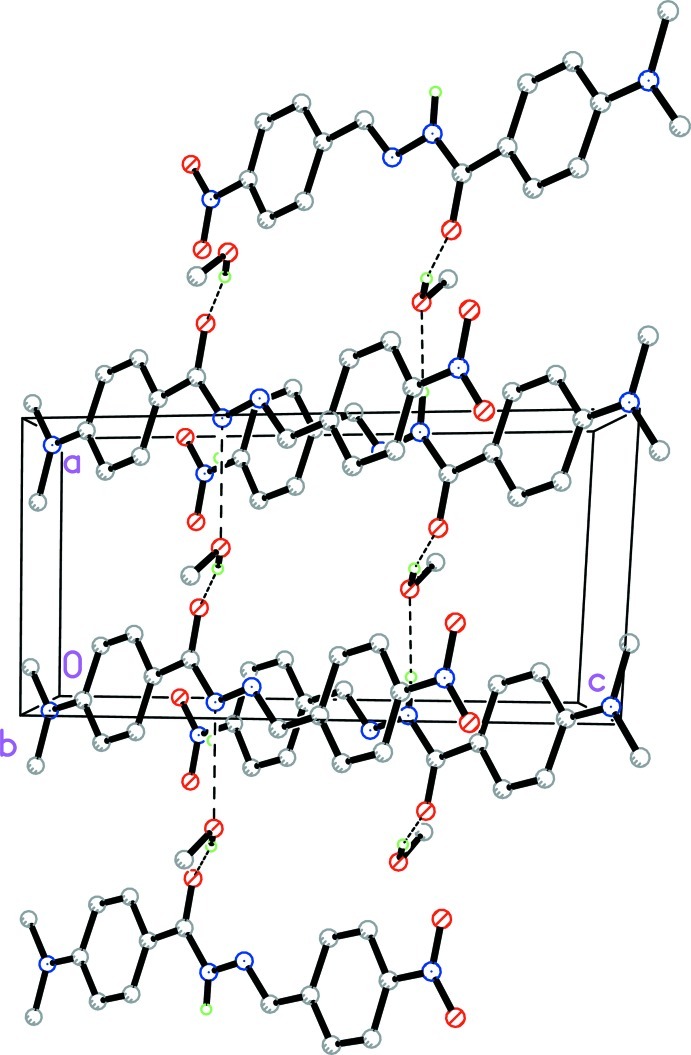
A portion of the crystal packing viewed down the b axis. Hydrogen bonds are drawn as dashed lines.

## supplementary crystallographic information

### Comment

Hydrazones derived from the condensation reactions of hydrazines with carbonyl-containing compounds have been found to possess many biological activities, such as antibacterial, anticonvulsant, anti-inflamatory, and antitubercular (Zhang *et al.*, 2012; Cacic *et al.*, 2006; Rauf *et al.*, 2008; Bedia *et al.*, 2006). Recently, a number of hydrazones have been prepared and structurally characterized (Horkaew *et al.*, 2012; Kargar *et al.*, 2012; Hu & Liu, 2012). As an extension of work on the structural characterization of hydrazones, the title compound is reported here.

The asymmetric unit of the title compound contains a hydrazone molecule and a methanol molecule of crystallization linked by O—H···O hydrogen bond (Fig. 1). The hydrazone molecule displays a *trans* conformation with respect to the C=N bond. Two aromatic rings in the hydrazone molecules form a dihedral angle of 0.4 (2)°. The nitro group is twisted from the attached benzene ring at 7.5 (2)°. Intermolecular N—H···O and O—H···O hydrogen bonds (Table 1) link alternating hydrazone and methanol molecules into chains in [100] (Fig. 2). The crystal packing exhibits π–π interactions between the aromatic ring from the neighbouring chains [centroid-centroid distances 3.734 (3), 3.903 (3) Å].

### Experimental

4-Nitrobenzaldehyde (0.1 mmol, 15.1 mg) and 4-dimethylaminobenzhydrazide (0.1 mmol, 17.9 mg) were stirred in 20 ml methanol at room temperature for 30 min. A large number of small and yellow single crystals were formed by slow evaporation of the methanolic solution containing the compound in air.

### Refinement

The amide H2 atom was located in a difference map and was refined isotropically, with restraint N—H = 0.90 (1) Å. The remaining H atoms were positioned geometrically and allowed to ride on their parent atoms, with C—H = 0.93-0.96 Å , O—H = 0.82 Å. The U_iso_ values were constrained to be 1.5U_eq_ of the carrier atom for methyl and hydroxyl H atoms and 1.2U_eq_ for the remaining H atoms. A rotating group model was used for the methyl groups.

### Crystal data

**Table:**

C_16_H_16_N_4_O_3_·CH_4_O	*Z* = 2
*M**_r_* = 344.37	*F*(000) = 364
Triclinic, *P*1	*D*_x_ = 1.318 Mg m^−^^3^
*a* = 6.6621 (12) Å	Mo *K*α radiation, λ = 0.71073 Å
*b* = 10.6685 (17) Å	Cell parameters from 2473 reflections
*c* = 13.3437 (13) Å	θ = 2.5–26.5°
α = 72.279 (2)°	µ = 0.10 mm^−^^1^
β = 83.444 (2)°	*T* = 298 K
γ = 73.984 (2)°	Block, yellow
*V* = 867.9 (2) Å^3^	0.30 × 0.27 × 0.23 mm

### Data collection

**Table:**

Bruker SMART CCD area-detector diffractometer	3140 independent reflections
Radiation source: fine-focus sealed tube	2082 reflections with *I* > 2σ(*I*)
Graphite monochromator	*R*_int_ = 0.035
ω scans	θ_max_ = 25.5°, θ_min_ = 1.6°
Absorption correction: multi-scan (*SADABS*; Sheldrick, 1996)	*h* = −8→8
*T*_min_ = 0.972, *T*_max_ = 0.978	*k* = −12→12
6198 measured reflections	*l* = −16→15

### Refinement

**Table:**

Refinement on *F*^2^	Primary atom site location: structure-invariant direct methods
Least-squares matrix: full	Secondary atom site location: difference Fourier map
*R*[*F*^2^ > 2σ(*F*^2^)] = 0.058	Hydrogen site location: inferred from neighbouring sites
*wR*(*F*^2^) = 0.208	H atoms treated by a mixture of independent and constrained refinement
*S* = 1.08	*w* = 1/[σ^2^(*F*_o_^2^) + (0.1182*P*)^2^ + 0.1132*P*] where *P* = (*F*_o_^2^ + 2*F*_c_^2^)/3
3140 reflections	(Δ/σ)_max_ < 0.001
233 parameters	Δρ_max_ = 0.32 e Å^−^^3^
1 restraint	Δρ_min_ = −0.40 e Å^−^^3^

### Special details

**Table:**

Geometry. All esds (except the esd in the dihedral angle between two l.s. planes) are estimated using the full covariance matrix. The cell esds are taken into account individually in the estimation of esds in distances, angles and torsion angles; correlations between esds in cell parameters are only used when they are defined by crystal symmetry. An approximate (isotropic) treatment of cell esds is used for estimating esds involving l.s. planes.
Refinement. Refinement of F^2^ against ALL reflections. The weighted R-factor wR and goodness of fit S are based on F^2^, conventional R-factors R are based on F, with F set to zero for negative F^2^. The threshold expression of F^2^ > 2sigma(F^2^) is used only for calculating R-factors(gt) etc. and is not relevant to the choice of reflections for refinement. R-factors based on F^2^ are statistically about twice as large as those based on F, and R- factors based on ALL data will be even larger.

### Fractional atomic coordinates and isotropic or equivalent isotropic displacement parameters (Å^2^)

**Table:**

	*x*	*y*	*z*	*U*_iso_*/*U*_eq_	
N1	0.0844 (3)	0.62271 (17)	0.39221 (13)	0.0471 (5)	
N2	0.0240 (3)	0.56468 (18)	0.32624 (14)	0.0478 (5)	
N3	0.1415 (4)	0.9440 (2)	0.71802 (16)	0.0655 (6)	
N4	−0.0400 (4)	0.2426 (2)	−0.00213 (16)	0.0715 (6)	
O1	0.3228 (3)	0.9304 (2)	0.7318 (2)	0.1055 (8)	
O2	−0.0001 (4)	1.0126 (2)	0.75895 (17)	0.0929 (7)	
O3	0.3593 (2)	0.47225 (17)	0.29021 (14)	0.0672 (5)	
O4	0.5727 (2)	0.63544 (19)	0.33104 (18)	0.0796 (6)	
H4	0.4999	0.5874	0.3254	0.119*	
C1	−0.0040 (3)	0.7521 (2)	0.51346 (15)	0.0427 (5)	
C2	0.2010 (3)	0.7245 (2)	0.54349 (16)	0.0484 (5)	
H2A	0.3067	0.6636	0.5177	0.058*	
C3	0.2480 (3)	0.7863 (2)	0.61062 (17)	0.0516 (6)	
H3	0.3848	0.7679	0.6307	0.062*	
C4	0.0892 (3)	0.8763 (2)	0.64797 (16)	0.0481 (5)	
C5	−0.1143 (3)	0.9036 (2)	0.62214 (17)	0.0520 (6)	
H5	−0.2194	0.9628	0.6498	0.062*	
C6	−0.1598 (3)	0.8413 (2)	0.55424 (17)	0.0508 (5)	
H6	−0.2973	0.8594	0.5354	0.061*	
C7	−0.0577 (3)	0.6877 (2)	0.44223 (17)	0.0486 (5)	
H7	−0.1971	0.6943	0.4334	0.058*	
C8	0.1735 (3)	0.4890 (2)	0.27631 (17)	0.0458 (5)	
C9	0.1067 (3)	0.4274 (2)	0.20565 (16)	0.0436 (5)	
C10	0.2582 (3)	0.3367 (2)	0.16454 (18)	0.0561 (6)	
H10	0.3963	0.3175	0.1828	0.067*	
C11	0.2113 (4)	0.2743 (3)	0.09784 (19)	0.0611 (6)	
H11	0.3172	0.2125	0.0730	0.073*	
C12	0.0067 (4)	0.3021 (2)	0.06654 (17)	0.0537 (6)	
C13	−0.1459 (3)	0.3920 (2)	0.10855 (17)	0.0547 (6)	
H13	−0.2842	0.4112	0.0904	0.066*	
C14	−0.0978 (3)	0.4533 (2)	0.17627 (17)	0.0495 (5)	
H14	−0.2038	0.5131	0.2029	0.059*	
C15	−0.2519 (5)	0.2624 (4)	−0.0273 (2)	0.0884 (9)	
H15A	−0.3265	0.2215	0.0343	0.133*	
H15B	−0.2541	0.2210	−0.0816	0.133*	
H15C	−0.3171	0.3581	−0.0515	0.133*	
C16	0.1216 (6)	0.1550 (4)	−0.0473 (3)	0.1070 (12)	
H16A	0.2272	0.2015	−0.0802	0.161*	
H16B	0.0640	0.1302	−0.0991	0.161*	
H16C	0.1825	0.0745	0.0068	0.161*	
C17	0.4766 (4)	0.7717 (3)	0.2827 (2)	0.0819 (8)	
H17A	0.3486	0.7993	0.3205	0.123*	
H17B	0.4468	0.7823	0.2114	0.123*	
H17C	0.5681	0.8271	0.2830	0.123*	
H2	−0.1140 (17)	0.579 (3)	0.324 (2)	0.080*	

### Atomic displacement parameters (Å^2^)

**Table:**

	*U*^11^	*U*^22^	*U*^33^	*U*^12^	*U*^13^	*U*^23^
N1	0.0436 (9)	0.0531 (10)	0.0547 (10)	−0.0173 (8)	−0.0043 (8)	−0.0244 (8)
N2	0.0375 (9)	0.0586 (11)	0.0595 (11)	−0.0156 (8)	−0.0030 (8)	−0.0311 (9)
N3	0.0835 (15)	0.0537 (12)	0.0673 (13)	−0.0147 (11)	−0.0210 (11)	−0.0247 (10)
N4	0.0817 (15)	0.0936 (16)	0.0658 (13)	−0.0425 (13)	0.0073 (11)	−0.0464 (12)
O1	0.0872 (15)	0.1166 (18)	0.146 (2)	−0.0217 (13)	−0.0391 (14)	−0.0764 (15)
O2	0.1084 (16)	0.0876 (14)	0.0976 (15)	−0.0057 (12)	−0.0188 (12)	−0.0597 (12)
O3	0.0373 (8)	0.0834 (12)	0.1006 (13)	−0.0109 (8)	−0.0093 (8)	−0.0561 (10)
O4	0.0382 (9)	0.0841 (13)	0.1399 (17)	−0.0125 (8)	−0.0084 (9)	−0.0664 (12)
C1	0.0416 (11)	0.0459 (11)	0.0455 (11)	−0.0149 (9)	−0.0015 (8)	−0.0166 (9)
C2	0.0425 (11)	0.0522 (12)	0.0542 (12)	−0.0093 (9)	−0.0034 (9)	−0.0223 (10)
C3	0.0445 (12)	0.0567 (13)	0.0575 (13)	−0.0133 (10)	−0.0108 (10)	−0.0183 (10)
C4	0.0586 (13)	0.0435 (11)	0.0468 (12)	−0.0157 (10)	−0.0077 (10)	−0.0150 (9)
C5	0.0523 (12)	0.0505 (12)	0.0554 (13)	−0.0082 (10)	0.0002 (10)	−0.0236 (10)
C6	0.0397 (11)	0.0573 (13)	0.0600 (13)	−0.0123 (10)	−0.0014 (9)	−0.0236 (10)
C7	0.0395 (11)	0.0558 (13)	0.0583 (13)	−0.0152 (10)	−0.0024 (9)	−0.0243 (10)
C8	0.0375 (11)	0.0487 (12)	0.0576 (13)	−0.0132 (9)	−0.0034 (9)	−0.0217 (10)
C9	0.0390 (10)	0.0490 (12)	0.0490 (11)	−0.0161 (9)	−0.0002 (8)	−0.0190 (9)
C10	0.0405 (11)	0.0689 (15)	0.0705 (15)	−0.0184 (10)	0.0038 (10)	−0.0346 (12)
C11	0.0551 (13)	0.0725 (16)	0.0718 (15)	−0.0232 (12)	0.0103 (11)	−0.0419 (13)
C12	0.0632 (14)	0.0644 (14)	0.0477 (12)	−0.0332 (12)	0.0039 (10)	−0.0232 (10)
C13	0.0489 (12)	0.0664 (14)	0.0578 (13)	−0.0211 (11)	−0.0083 (10)	−0.0223 (11)
C14	0.0416 (11)	0.0569 (13)	0.0572 (13)	−0.0147 (10)	−0.0025 (9)	−0.0245 (10)
C15	0.097 (2)	0.120 (2)	0.0789 (19)	−0.0550 (19)	−0.0136 (16)	−0.0449 (17)
C16	0.112 (3)	0.148 (3)	0.112 (3)	−0.066 (2)	0.034 (2)	−0.095 (3)
C17	0.0672 (16)	0.101 (2)	0.085 (2)	−0.0297 (16)	−0.0022 (14)	−0.0310 (17)

### Geometric parameters (Å, º)

**Table:**

N1—C7	1.264 (3)	C6—H6	0.9300
N1—N2	1.368 (2)	C7—H7	0.9300
N2—C8	1.349 (3)	C8—C9	1.468 (3)
N2—H2	0.892 (10)	C9—C10	1.383 (3)
N3—O1	1.205 (3)	C9—C14	1.390 (3)
N3—O2	1.212 (3)	C10—C11	1.368 (3)
N3—C4	1.464 (3)	C10—H10	0.9300
N4—C12	1.367 (3)	C11—C12	1.398 (3)
N4—C16	1.427 (4)	C11—H11	0.9300
N4—C15	1.432 (3)	C12—C13	1.388 (3)
O3—C8	1.228 (2)	C13—C14	1.374 (3)
O4—C17	1.396 (3)	C13—H13	0.9300
O4—H4	0.8200	C14—H14	0.9300
C1—C6	1.386 (3)	C15—H15A	0.9600
C1—C2	1.394 (3)	C15—H15B	0.9600
C1—C7	1.454 (3)	C15—H15C	0.9600
C2—C3	1.368 (3)	C16—H16A	0.9600
C2—H2A	0.9300	C16—H16B	0.9600
C3—C4	1.378 (3)	C16—H16C	0.9600
C3—H3	0.9300	C17—H17A	0.9600
C4—C5	1.367 (3)	C17—H17B	0.9600
C5—C6	1.376 (3)	C17—H17C	0.9600
C5—H5	0.9300		
			
C7—N1—N2	117.48 (17)	C10—C9—C8	117.77 (18)
C8—N2—N1	118.39 (17)	C14—C9—C8	125.07 (18)
C8—N2—H2	126.9 (17)	C11—C10—C9	122.1 (2)
N1—N2—H2	114.6 (17)	C11—C10—H10	119.0
O1—N3—O2	122.8 (2)	C9—C10—H10	119.0
O1—N3—C4	118.8 (2)	C10—C11—C12	120.9 (2)
O2—N3—C4	118.4 (2)	C10—C11—H11	119.5
C12—N4—C16	120.4 (2)	C12—C11—H11	119.5
C12—N4—C15	121.0 (2)	N4—C12—C13	121.8 (2)
C16—N4—C15	118.5 (2)	N4—C12—C11	121.1 (2)
C17—O4—H4	109.5	C13—C12—C11	117.08 (19)
C6—C1—C2	118.84 (19)	C14—C13—C12	121.61 (19)
C6—C1—C7	119.61 (19)	C14—C13—H13	119.2
C2—C1—C7	121.54 (18)	C12—C13—H13	119.2
C3—C2—C1	120.56 (19)	C13—C14—C9	121.15 (19)
C3—C2—H2A	119.7	C13—C14—H14	119.4
C1—C2—H2A	119.7	C9—C14—H14	119.4
C2—C3—C4	118.83 (19)	N4—C15—H15A	109.5
C2—C3—H3	120.6	N4—C15—H15B	109.5
C4—C3—H3	120.6	H15A—C15—H15B	109.5
C5—C4—C3	122.28 (19)	N4—C15—H15C	109.5
C5—C4—N3	119.25 (19)	H15A—C15—H15C	109.5
C3—C4—N3	118.5 (2)	H15B—C15—H15C	109.5
C4—C5—C6	118.42 (19)	N4—C16—H16A	109.5
C4—C5—H5	120.8	N4—C16—H16B	109.5
C6—C5—H5	120.8	H16A—C16—H16B	109.5
C5—C6—C1	121.0 (2)	N4—C16—H16C	109.5
C5—C6—H6	119.5	H16A—C16—H16C	109.5
C1—C6—H6	119.5	H16B—C16—H16C	109.5
N1—C7—C1	120.24 (19)	O4—C17—H17A	109.5
N1—C7—H7	119.9	O4—C17—H17B	109.5
C1—C7—H7	119.9	H17A—C17—H17B	109.5
O3—C8—N2	120.71 (18)	O4—C17—H17C	109.5
O3—C8—C9	121.40 (18)	H17A—C17—H17C	109.5
N2—C8—C9	117.89 (17)	H17B—C17—H17C	109.5
C10—C9—C14	117.15 (18)		

### Hydrogen-bond geometry (Å, º)

**Table:**

*D*—H···*A*	*D*—H	H···*A*	*D*···*A*	*D*—H···*A*
O4—H4···O3	0.82	1.92	2.733 (2)	170
N2—H2···O4^i^	0.89 (1)	2.01 (1)	2.889 (2)	170 (2)

Symmetry code: (i) *x*−1, *y*, *z*.
